# Efgartigimod as a fast-acting treatment in generalized very-late-onset myasthenia gravis

**DOI:** 10.3389/fimmu.2025.1579859

**Published:** 2025-04-17

**Authors:** Zhouao Zhang, Mingjin Yang, Xinyan Guo, Tianyu Ma, Zhouyi Wang, Tiancheng Luo, Deyou Peng, Xue Du, Xiaoyu Huang, Yong Zhang

**Affiliations:** ^1^ Department of Neurology, The Affiliated Hospital of Xuzhou Medical University, Xuzhou, Jiangsu, China; ^2^ First Clinical Medical College, Xuzhou Medical University, Xuzhou, Jiangsu, China; ^3^ Central Laboratory, The Affiliated Hospital of Xuzhou Medical University, Xuzhou, Jiangsu, China

**Keywords:** very-late-onset myasthenia gravis, fast-acting treatment, efgartigimod, MG-ADL, clinical meaningful improvement

## Abstract

**Objective:**

Efgartigimod (EFG), a neonatal Fc receptor antagonist that facilitates the degradation of pathogenic immunoglobulin G, is approved for the treatment of generalized myasthenia gravis (MG). This study aims to evaluate the efficacy and safety of EFG in patients with very-late-onset myasthenia gravis (VLOMG).

**Methods:**

This study enrolled 15 consecutive patients diagnosed with VLOMG who received EFG treatment. Baseline demographic and clinical characteristics, as well as dynamic changes in the MG-specific activities of daily living (MG-ADL) score and quantitative MG (QMG) score, were systematically recorded.

**Results:**

Patients were stratified into two groups: a worse group (n = 8) and a new-diagnosed group (n = 7), the latter of which included 5 patients who had received monotherapy with pyridostigmine (Py) prior to EFG. At week 5, the mean changes in MG-ADL scores were -4.9 ± 3.3 in the overall VLOMG cohort, -6.1 ± 3.1 in the new-diagnosed group, -6.6 ± 3.6 in the mono-Py subgroup, and -3.8 ± 3.2 in the worse group. The clinical meaningful improvement (CMI) rate was 86.7% (13/15) in the overall cohort, 75.0% (6/8) in the worse group, and 100.0% (7/7) in the new-diagnosed group. During a mean follow-up time of 39.2 ± 16.2 weeks, symptoms remained stable in responsive patients, with various treatment strategies implemented following the fast-acting treatment of EFG. No adverse drug reactions were reported in this cohort.

**Conclusion:**

This study demonstrates that EFG is an effective and safe treatment for patients with VLOMG. EFG exhibits potential as an early, fast-acting treatment and may confer sustained clinical benefits in this patient population.

## Introduction

Myasthenia gravis (MG) is a prototypical autoimmune disorder characterized by the production of autoantibodies directed against key neuromuscular junction components, including acetylcholine receptors (AChR), muscle-specific tyrosine kinase (MuSK), and other postsynaptic membrane-associated proteins (1). The typical symptom of MG was fluctuating fatigue of skeletal muscles (1). MG demonstrates significant clinical heterogeneity and can be categorized into distinct subgroups according to antibody profiles, age at onset, predominant symptoms, and thymic pathology (2). Therefore, the development of a treatment strategy for MG is challenging and requires a personalized approach.

Patients with onset-age older than 50 years old are classified as late-onset MG (LOMG) (3). In recent years, the prevalence of LOMG has increased significantly, likely due to improved disease recognition and the aging population (4, 5). Consequently, recent studies have proposed further stratification of LOMG into two subgroups, with very-late-onset myasthenia gravis (VLOMG) defined by symptom onset after 65 years of age (6). Patients with VLOMG typically present with more severe initial symptoms, an elevated risk of disease exacerbation, but have better long-term outcomes (7, 8). Furthermore, VLOMG patients frequently exhibit multiple comorbidities, including cerebrovascular disease, diabetes mellitus, chronic renal failure, and osteoporosis, which may obscure the early diagnosis of MG and substantially limit the use of corticosteroids and other immunosuppressive therapies (9, 10). Therefore, early initiation of fast-acting therapies with favorable safety is critical for this patient population.

Early fast-acting treatment is characterized by the administration of intensive therapeutic interventions during the initial disease phase to rapidly achieve minimal manifestation status (MMS), typically involving high-dose intravenous methylprednisolone (IVMP), plasma exchange (PLEX), and intravenous immunoglobulin (IVIg) (11, 12). However, the utilization of these treatments in VLOMG patients is often limited by significant clinical constraints. For example, IVMP is associated with adverse effects such as hyperglycemia and peptic ulcers, while IVIg may increase the risk of thromboembolic events and acute renal failure, particularly in elderly patients (13). Recently, neonatal Fc receptor (FcRn) inhibitors, which is praised as “plasma exchange in a bottle”, dramatically revolutionizes the treatment landscape of MG (14). Efgartigimod (EFG) is a human IgG1 Fc-fragment that competitively inhibits the binding of endogenous IgG to FcRn, thereby accelerating the degradation of pathogenic IgG antibodies (15). In a phase 3 clinical trial, EFG demonstrated rapid efficacy in AChR antibody-positive generalized MG (gMG), with clinical improvement observed within the first treatment cycle and a response onset within 2 weeks in 87% of responders (15). In addition, multiple studies have highlighted the efficacy of EFG as a fast-acting therapeutic option for myasthenic crisis (MC) or impending MC (16–19). Moreover, EFG is generally well-tolerated in gMG patients (15, 20). Given the fast efficacy and safety of EFG, it has the potential to be a fast-acting strategy for VLOMG patients. Nevertheless, there has been a lack of research specifically investigating the application of EFG within this MG subgroup. Therefore, we conducted a retrospective study to evaluate the efficacy of EFG in VLOMG patients and its potential role in new-diagnosed patients.

## Materials and methods

### Study population

This retrospective and pilot case series study consecutively recruited 15 patients with generalized VLOMG at the Department of Neurology of the Affiliated Hospital of Xuzhou Medical University from November 2023 to December 2024. The diagnosis of MG was affirmed based on typical clinical symptoms, which are characterized by fluctuating skeletal muscle weakness, along with at least one positive auxiliary test such as neostigmine test, repetitive nerve stimulation, and serological antibodies detection. Patients with one or more of the following conditions were excluded: (1) incomplete baseline records; (2) other autoimmune diseases; (3) history of B-cell targeted monoclonal drugs; (4) therapies adjustment within 5 weeks after the first EFG infusion; (5) MG-specific activities of daily living (MG-ADL) score ≤ 5 points. Patients with VLOMG were classified into two groups according to the disease status at admission: new-diagnosed group (n = 7) and worse group (n = 8). The status of worse was diagnosed in accordance with Myasthenia Gravis Foundation of America (MGFA) post-intervention status (PIS) and was defined as a minimum increase of ≥5 points in quantitative myasthenia gravis (QMG) score or ≥2 points in MG-ADL score from the previous visit (21, 22). In addition, to exclude the effect of pyridostigmine (Py) on the efficacy observation, only new-diagnosed patients whose symptoms did not improve after Py were included.

### Data collection

Demographic data, including age, gender, disease duration, antibody profile, thymic status, and comorbidities, were gathered from medical records. Blood samples were collected on an empty stomach and the concentrations of serum IgG were measured by immunonephelometric before infusion of EFG. The severity of MG was evaluated by MG-ADL score, QMG score, and MGFA classification. Each QMG score was evaluated more than 8 hours after the last use of Py (23). In addition, patient-reported adverse events were collected from the interviews.

### Statistical analysis

Statistical Package for the Social Sciences (SPSS 26.0) and GraphPad Prism software 9.2.0 were utilized for statistical analysis. Categorical variables were expressed as numbers (percentages), normally distributed variables were expressed as mean ± standard deviation (SD), and non-normally distributed variables were expressed as median (interquartile range). Independent data was compared by unpaired T-test (normal distribution) or Mann–Whitney U test (abnormal distribution) and paired data was compared by paired T-test or Wilcoxon matched-pairs test. The statistical significance was considered as a p value of < 0.05 (two tailed).

## Results

### Baseline characteristic of study population

This study included 15 patients with generalized VLOMG according to inclusion and exclusion criteria. The detailed clinical information of those patients was listed in **Table 1**. The mean age was 73.9 ± 5.9 years old, and the female-to-male ratio was 8:7, with a median duration of 12.0 (2.0, 33.0) months and a mean follow-up time of 39.2 ± 16.2 weeks. All patients tested positive for anti-AChR antibody and 3/15 (20.0%) of patients had thymoma. The distribution of MGFA classification was as follows: IIa (3/15, 20.0%), IIIa (9/15, 60.0%), and IIIb (3/15, 20.0%). The therapies before EFG included pyridostigmine (15/15, 100%), prednisone (7/15, 46.7%), mycophenolate mofetil (2/15, 13.3%), and tacrolimus (2/15, 13.3%). The comorbidities were documented and listed as follows: hypertension (9/15, 60.0%), stroke (4/15, 26.7%), coronary artery disease (2/15, 13.3%), renal failure (2/15, 13.3%), diabetes (3/15, 20.0%), heart failure (1/15, 6.7%), and atrial fibrillation (1/15, 6.7%).

**Table 1:** Basic clinical characteristics and therapies prior to the efgartigimod of the generalized VLOMG cohort.

Patient No.	Sex	Age, years	Duration, months	Antibody	Thymoma	Comorbidities	Previous therapies	Disease state	MGFA classification	MG-ADL score	QMG score	Follow-up, weeks
1	F	69	1	AChR	Y	None.	Py, P, TAC	New	3a	5	12	61
2	M	75	49	AChR	N	Hypertension, renal failure	Py, P	Worse	3a	5	14	63
3	F	76	12	AChR	N	Hypertension	Py, P	Worse	3a	9	19	53
4	M	68	4	AChR	Y	None.	Py	New	3a	6	15	52
5	F	66	8	AChR	Y	Hypertension	Py	Worse	3a	9	9	53
6	M	69	38	AChR	N	Hypertension	Py, P, MMF	Worse	3b	12	18	48
7	F	86	2	AChR	N	Coronary artery disease	Py	New	3a	6	17	43
8	M	76	8	AChR	N	Hypertension, stroke	Py	Worse	2a	6	10	46
9	F	70	55	AChR	N	Hypertension, diabetes, coronary artery	Py	Worse	2a	5	8	35
10	F	73	33	AChR	N	Hypertension, diabetes, stroke, Parkinson’s disease	Py, P, TAC	Worse	3a	12	14	32
11	M	67	13	AChR	N	None.	Py	New	2a	7	14	27
12	M	83	1	AChR	N	Ischemic stroke	Py, P	New	3b	12	18	23
13	F	76	12	AChR	N	Hypertension, renal failure, heart failure, atrial fibrillation	Py, P, MMF	Worse	3a	8	16	21
14	M	79	1	AChR	N	Hypertension	Py	New	3b	10	16	16
15	F	76	6	AChR	N	Hypertension, diabetes	Py	New	3a	9	9	15

AChR, acetylcholine receptor; EFG, Efgartigimod; MG-ADL, myasthenia gravis-specific activities of daily living; MGFA, Myasthenia Gravis Foundation of America; MMF, mycophenolate mofetil; P, prednisone; Py, pyridostigmine; QMG, quantitative myasthenia gravis; TAC, tacrolimus; VLOMG, very-late-onset myasthenia gravis.

### Clinical response to the first cycle of efgartigimod treatment

**Figure 1:**
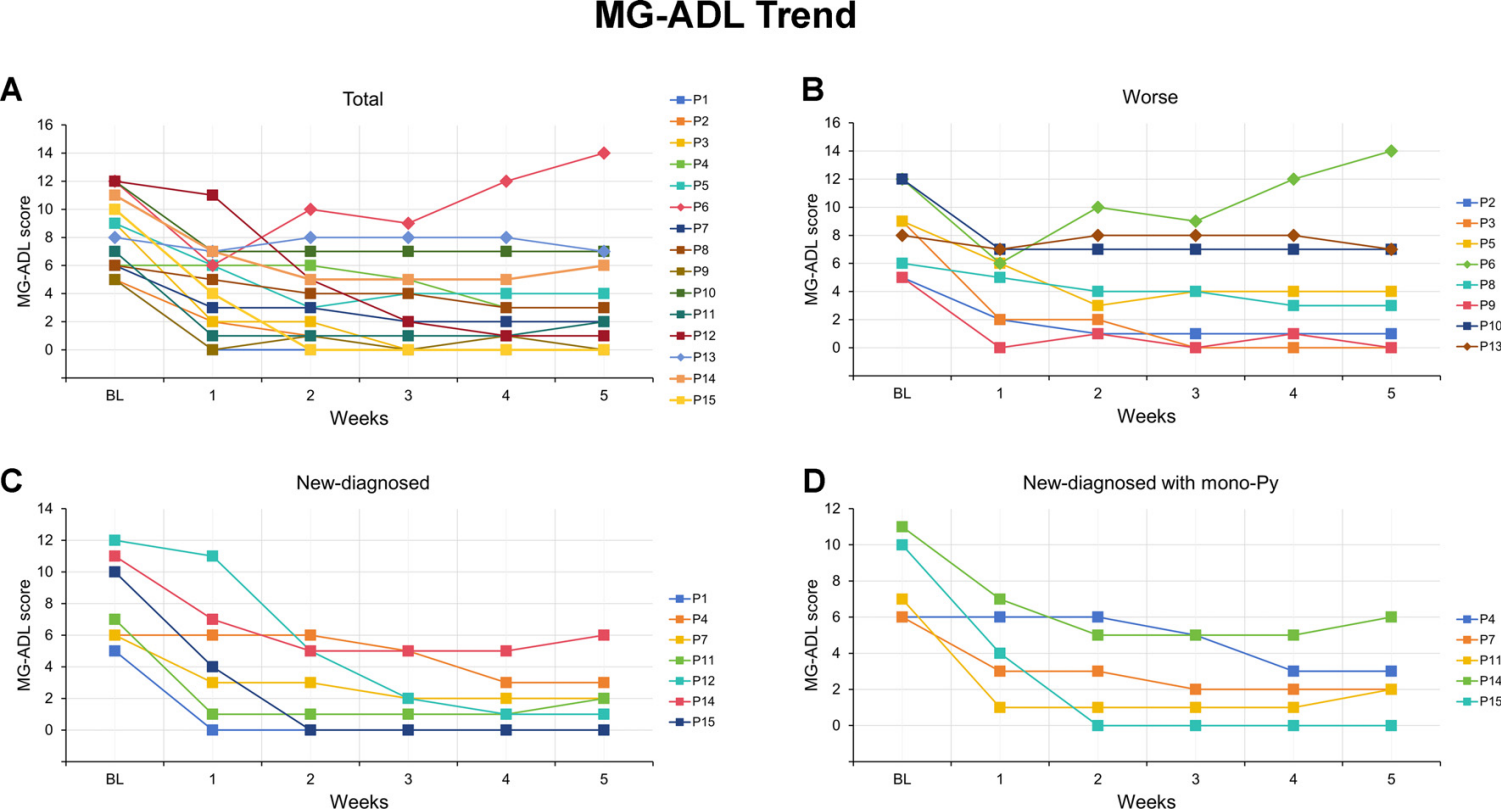
MG-ADL scores for each of the VLOMG patients from baseline to week 5 after efgartigimod. **(A)** Detailed changes in MG-ADL score for each of the 15 VLOMG patients; **(B)** Detailed changes in MG-ADL score for each of the 8 VLOMG patients in worse group; **(C)** Detailed changes in MG-ADL score for each of the 7 VLOMG patients in new-diagnosed group; **(D)** Detailed changes in MG-ADL score for each of the 5 VLOMG patients in new-diagnosed with mono-Py group MG-ADL, myasthenia gravis-specific activities of daily living; P, patient; Py, pyridostigmine; VLOMG, very-late-onset myasthenia gravis.

**Figure 2:**
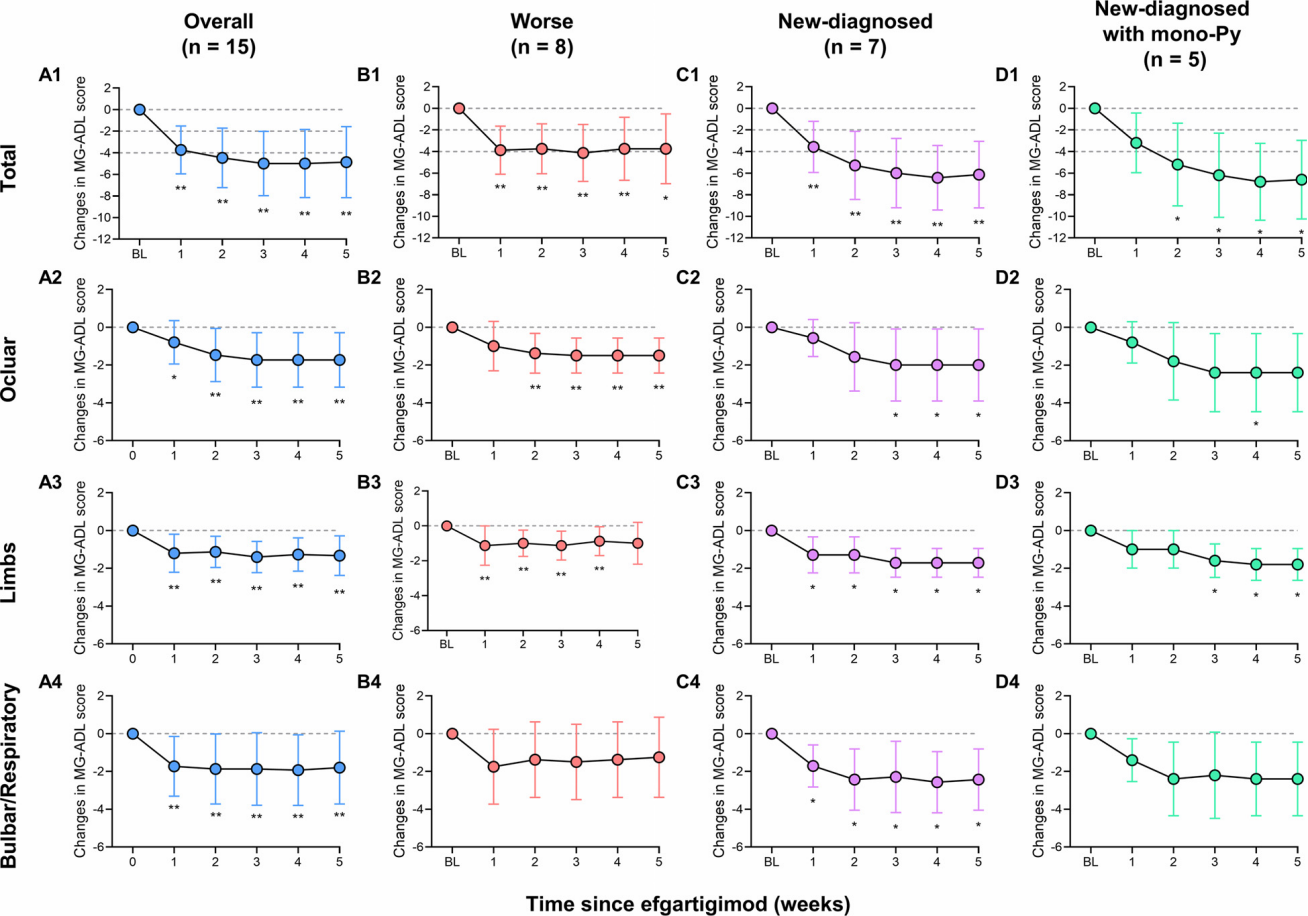
Mean changes in MG-ADL score and MG-ADL subdomains in patients with VLOMG. **(A1–A4)** Mean changes in MG-ADL score and MG-ADL subdomains in total VLOMG patients; **(B1–B4)** Mean changes in MG-ADL score and MG-ADL subdomains in worse group; **(C1–C4)** Mean changes in MG-ADL score and MG-ADL subdomains in new-diagnosed group; **(D1–D4)** Mean changes in MG-ADL score and MG-ADL subdomains in new-diagnosed with mono-Py group. *p < 0.05, **p < 0.01. MG-ADL, myasthenia gravis-specific activities of daily living; Py, pyridostigmine; VLOMG, very-late-onset myasthenia gravis.

**Figure 3:**
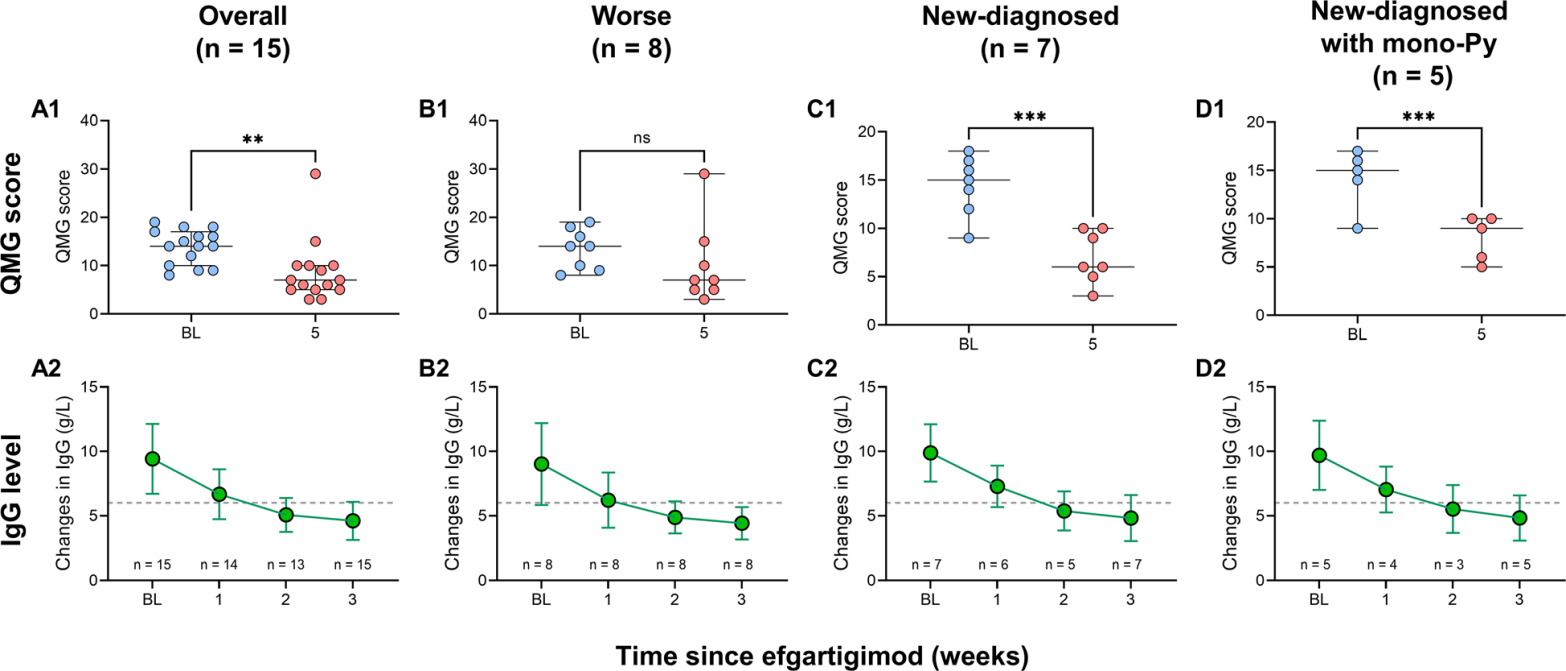
Changes in QMG score and serum IgG levels in patients with VLOMG. **(A1–D1)** Comparison of QMG score between baseline and week 5 in VLOMG patients and different subgroups of VLOMG patients; **(A2–D2)** Mean changes in serum IgG from baseline to week 3 in VLOMG patients and different subgroups of VLOMG patients. **p < 0.01, ***p < 0.001; ns, no significance. IgG, immunoglobulin G; Py, pyridostigmine; QMG, quantitative myasthenia gravis; VLOMG, very-late-onset myasthenia gravis.

All patients received at least one cycle of EFG treatment on schedule (10 mg/kg, 4 consecutive weeks). In this cohort, baseline QMG score was 13.9 ± 3.6 and baseline MG-ADL score was 8.2 ± 2.7. The detailed changes in MG-ADL score trend from baseline to week 5 were presented in **Figure 1**. The total MG-ADL score of all patients significantly decreased since week 1: -3.7 ± 2.2 by week 1 and -4.9 ± 3.3 by week 5 (both p = 0.001, **Figure 2A1**). In addition, the mean score changes by week 1 in MG-ADL subdomains were as follows: ocular: -0.8 ± 1.2; limbs: -1.2 ± 1.0; bulbar/respiratory: -1.7 ± 1.6 (all p < 0.05, **Figures 2A2–A4**). By week 5, the changes in the MG-ADL scores for ocular, limb, and bulbar/respiratory functions were -1.7 ± 1.4, -1.3 ± 1.0, and -1.8 ± 1.9, respectively (all p < 0.01, **Figures 2A2–A4**). The QMG score was compared between the baseline and week 5. The QMG score was 7.0 (5.0, 10.0) in all patients by week 5 and was significantly lower than at baseline (p = 0.005, **Figure 3A1**). Besides, the baseline level of IgG was 9.6 ± 2.7 g/L, and it was reduced rapidly by 30.2 ± 9.4% by week 1 (6.7 ± 1.9 g/L) and 49.9 ± 13.5% by week 3 (4.7 ± 1.5 g/L) (**Figure 3A2**).

**Figure 4:**
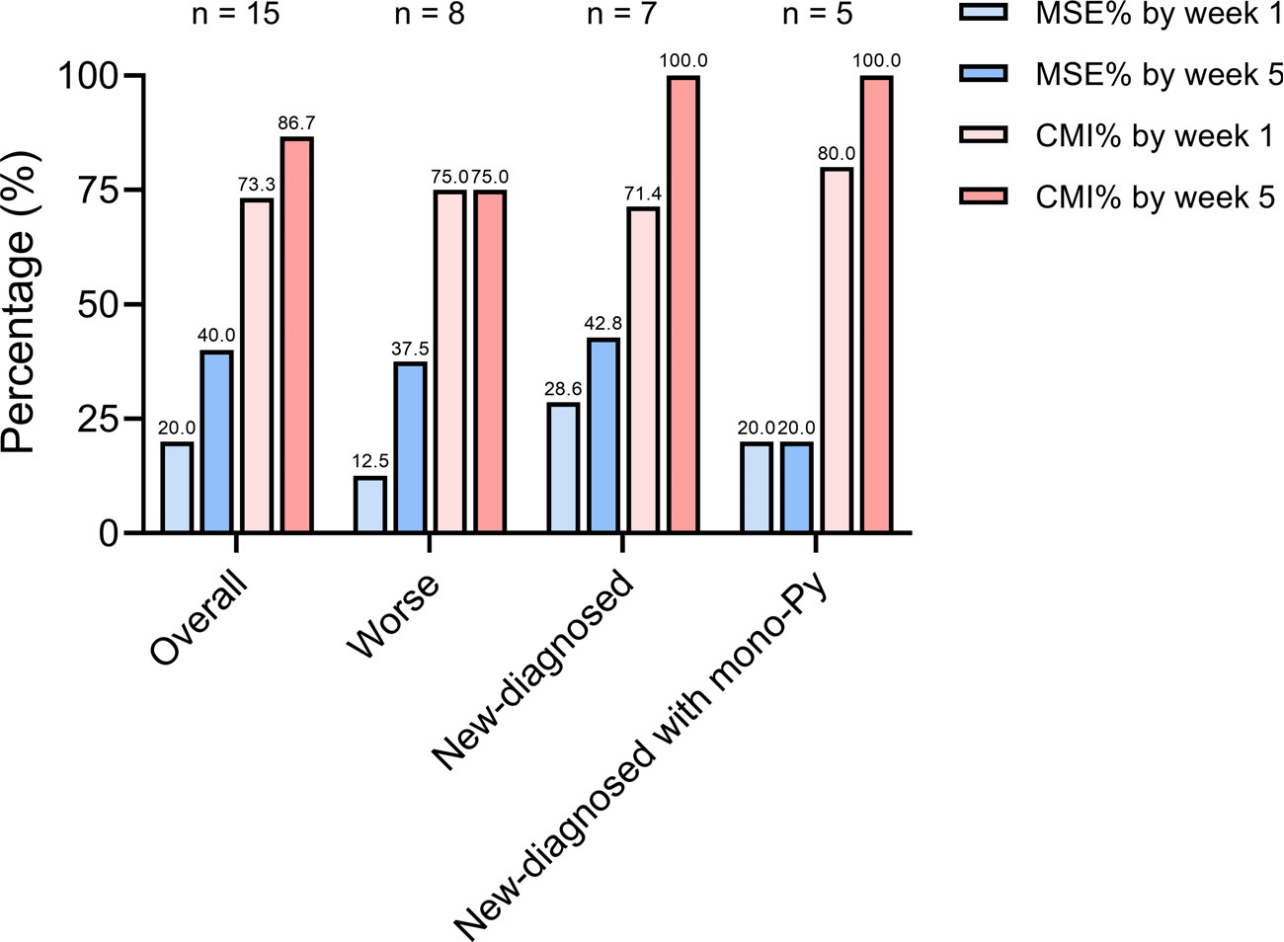
The proportion of VLOMG patients with different post-intervention status. CMI, clinical meaningful improvement; MSE, minimal symptom expression; Py, pyridostigmine; VLOMG, very-late-onset myasthenia gravis.

We stratified the patients by status after EFG according to MG-ADL score. Clinical meaningful improvement (CMI) was defined as a reduction of ≥2 points in the MG-ADL score from baseline and minimal symptom expression (MSE) was defined as an MG-ADL score of 0 or 1 (21, 24). The proportion of CMI in all patients was 73.3% by week 1 and reached 86.7% by week 5 (**Figure 4**). In addition, 3/15 (20.0%) of patients rapidly achieved MSE by week 1 and 6/15 (40.0%) of patients achieved MSE by week 5. No patient-reported adverse event, such as infections, reduction of albumin, was reported in this cohort. Furthermore, renal function was assessed in two patients suffering from renal failure, and no adverse reactions were noted either.

### Efgartigimod in VLOMG patients with different status at admission

Patients were categorized into new-diagnosed group (n = 7) and worse group (n= 8) based on admission status. No significant differences were observed between the two groups regarding gender, age, MGFA classification, thymic status, previous treatments, baseline MG-ADL score, and QMG score, except for disease duration (all p > 0.05, **Table 2**). The detailed changes in MG-ADL score were shown in **Figures 1B, C**. The baseline MG-ADL score was 8.2 ± 2.8 in the worse group and the changes in MG-ADL score were -3.9 ± 2.5 by week 1 (p = 0.002, **Figure 2B1**) and -3.8 ± 3.2 by week 5 (p = 0.014, **Figure 2B1**) compared with baseline. In the new-diagnosed group, the baseline MG-ADL score was 8.1 ± 2.8, and the changes were -3.6 ± 2.4 by week 1 (p = 0.007, **Figure 2C1**) and -6.1 ± 3.1 by week 5 (p = 0.002, **Figure 2C1**). There was no significant difference in weekly changes of MG-ADL scores between the two groups (all p > 0.05, **Table 2**). The changes in MG-ADL subdomains of those two groups were presented in **Figures 2B1–C4**. By week 1, changes in MG-ADL scores for ocular, limb, and bulbar/respiratory symptoms were -1.0 ± 1.3 (p = 0.068), -1.1 ± 1.1 (p = 0.037), and -1.8 ± 2.0 (p = 0.055) in the worse group, and -0.6 ± 1.0 (p = 0.172), -1.3 ± 1.0 (p = 0.034), and -1.7 ± 1.1 (p = 0.026) in the new-diagnosed group. By week 5, these changes were -1.5 ± 1.9 (p = 0.003), -1.0 ± 1.2 (p = 0.052), and -1.2 ± 2.1 (p = 0.135) in the worse group, and -2.0 ± 1.9 (p = 0.033), -1.7 ± 0.8 (p = 0.016), and -2.4 ± 1.6 (p = 0.026) in the new-diagnosed group. Additionally, five newly diagnosed patients with a baseline total MG-ADL score of 8.2 ± 2.7 received Py treatment alone before EFG. Changes in MG-ADL scores from baseline to week 5 are shown in **Figures 1D** and **2D1–D4**. Post-treatment, MG-ADL scores decreased by -3.2 ± 2.8 (p = 0.061) at week 1 and further declined by -6.6 ± 3.6 (p = 0.016) at week 5. Analysis by muscle groups revealed the following trends: at week 1, ocular scores decreased by -0.8 ± 1.1 (p = 0.178), limb scores by -1.0 ± 1.0 (p = 0.089), and bulbar/respiratory scores by -1.4 ± 1.1 (p = 0.102). By week 5, ocular scores decreased by -2.4 ± 2.1 (p = 0.061), limb scores by -1.8 ± 0.8 (p = 0.041), and bulbar/respiratory scores by -2.4 ± 2.0 (p = 0.066).

**Table 2:** Baseline clinical features and mean changes in MG-ADL scores for two subgroups of VLOMG.

	New-diagnosed (n = 7)	Worse (n = 8)	t/z/x^2^	P value
Female, n (%)	3 (42.8)	5 (62.5)	0.582	0.619
Age, years (SD)	75.4 ± 7.6	72.6 ± 3.8	-0.879	0.403
Duration, months	2.0 (1.0, 6.0)	26.5 (12.0, 46.2)	2.906	0.002
MGFA classification, II:III, n	1:6	2:6	0.273	1.000
Thymoma, n (%)	2 (28.5)	1 (12.5)	0.608	0.569
Previous therapies				
Pyridostigmine, n (%)	7 (100.0)	8 (100.0)	-	-
Prednisone, n (%)	2 (28.6)	5 (62.5)	1.761	0.315
Any NSIST, n (%)	1 (14.3)	3 (37.5)	1.071	0.569
QMG score (SD)	14.4 ± 3.1	13.5 ± 4.1	-0.485	0.636
MG-ADL (SD)	8.1 ± 2.8	8.2 ± 2.8	0.074	0.942
Changes in MG-ADL				
Week 1	-3.6 ± 2.4	-3.9 ± 2.2	0.255	0.802
Week 2	-5.3 ± 3.1	-3.8 ± 2.3	-1.087	0.297
Week 3	-6.0 ± 3.2	-4.1 ± 2.6	-1.241	0.237
Week 4	-6.4 ± 3.0	-3.8 ± 2.9	-1.754	0.103
Week 5	-6.1 ± 3.1	-3.8 ± 3.2	-1.460	0.168

MG-ADL, myasthenia gravis-specific activities of daily living; MG, myasthenia gravis; NISIST, non-steroidal immunosuppressive therapy; VLOMG, very-late-onset myasthenia gravis.

The comparison of QMG scores between baseline and week 5 showed significant reductions in both groups: from 14.4 ± 3.1 to 7.0 ± 2.7 (p < 0.001, **Figure 3C1**) in the new-diagnosed group, and from 14.2 ± 3.1 to 8.0 ± 2.3 (p < 0.001, **Figure 3D1**) in the new-diagnosed with mono-Py group. Although the QMG score in the worse group decreased from 13.5 ± 4.1 to 7.0 (5.0, 13.8), this reduction did not reach statistical significance (p = 0.106), which may be attributed to the small sample size. In addition, the levels of serum IgG simultaneously decreased in three subgroups (**Figures 3B2–D2**).

The CMI rate in the new-diagnosed group (5/7, 71.4%) was slightly lower than that in the worse group (6/8, 75.0%) at week 1. However, by week 5, the CMI rate in the new-diagnosed group increased to 7/7 (100%), surpassing that of the worse group (6/8, 75.0%) (**Figure 4**). At week 5, the CMI rates were as follows: 3/8 (37.5%) in the worse group, 3/7 (42.8%) in the new-diagnosed group, and 1/5 (20.0%) in the new-diagnosed with mono-Py group (**Figure 4**).

### Sequential therapies after the first cycle of efgartigimod treatment

**Figure 5:**
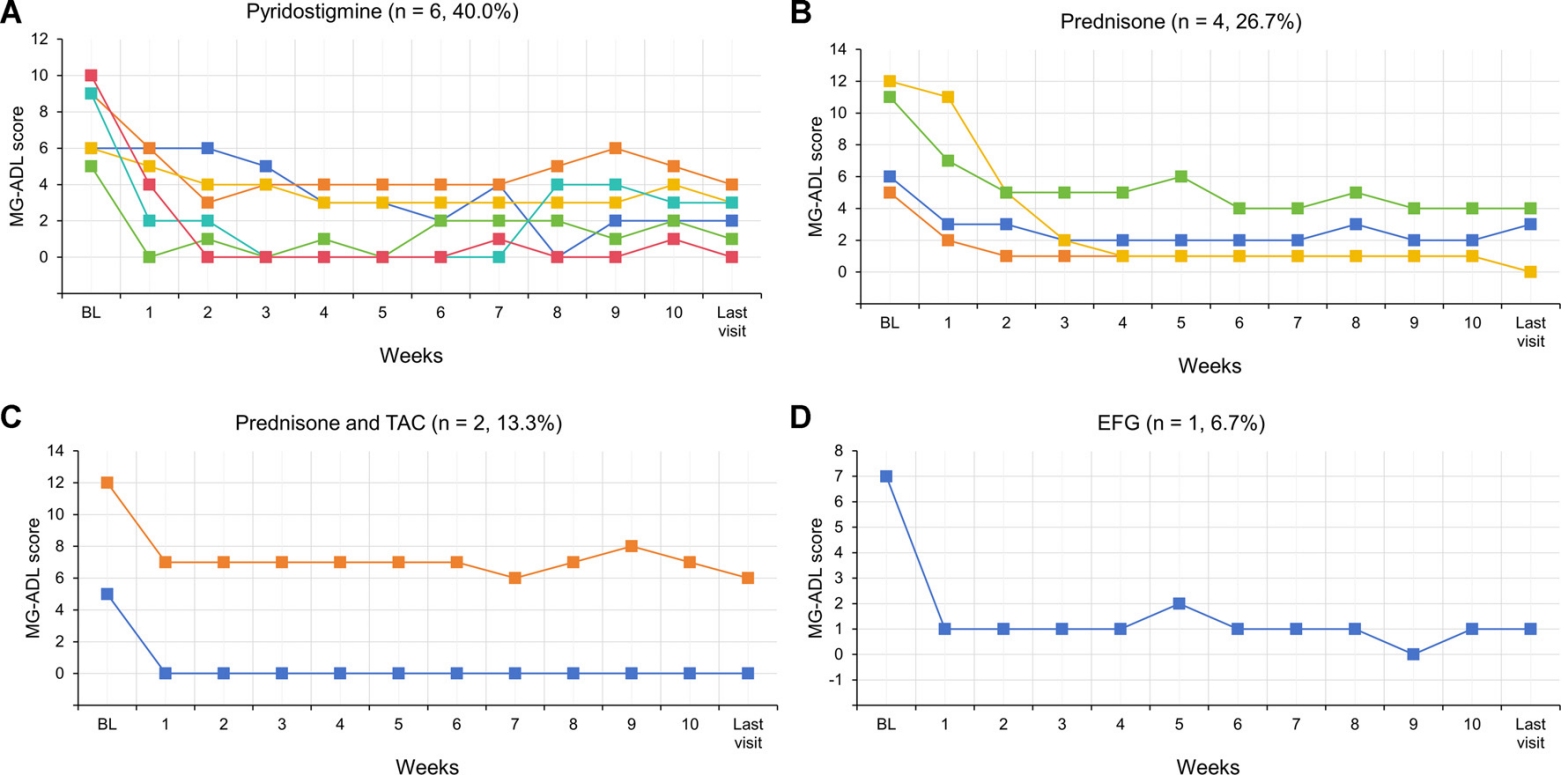
Dynamic changes in MG-ADL scores for each of 13 responded VLOMG patients from baseline to the last visit. EFG, Efgartigimod; MG-ADL, myasthenia gravis-specific activities of daily living; Py, pyridostigmine; TAC, tacrolimus; VLOMG, very-late-onset myasthenia gravis.

The therapeutic strategies following five weeks of initial EFG treatment were distributed as follows: 6/15 patients (40.0%) received Py monotherapy; 4/15 patients (26.7%) were administered oral prednisone; 2/15 patients (13.3%) received combined prednisone and tacrolimus; one patient continued EFG treatment at a regular frequency (every two to three weeks); and the remaining two patients, who were non-responders, were transitioned to complement inhibitor. The longer-term follow-up of changes in MG-ADL scores for 13 responded patients were showed in **Figure 5**. As presented, regardless of subsequent treatment regimens, both the MG-ADL scores at week 10 and at the last visit showed significant reductions compared to baseline.

### Clinical profiles of patients with poor response to efgartigimod

In this cohort, 2/15 (13.3%) patients did not achieve CMI in the first cycle of EFG. The detailed clinical profiles of these patients were presented as follows:

Patient 6 was a 69-year-old male with a history of hypertension and two previous MC prior to admission. Following the most recent crisis, the patient had been maintained on long-term oral glucocorticoids, Py, and mycophenolate mofetil. At baseline, his MG-ADL score was 12 points, QMG score was 18 points, and MGFA classification was 3b. Initial improvement was observed on day 7 after EFG, with the MG-ADL score decreasing to 7 points. However, despite receiving 4 consecutive EFG infusions, his clinical condition deteriorated, with a rapid increase in MG-ADL score culminating in a MC on day 55. Following this event, the treatment regimen was switched to complement inhibitors, resulting in gradual clinical improvement. The patient subsequently received maintenance therapy with complement inhibitors every two weeks, during which his MG-ADL score remained stable, fluctuating between 1 and 3 points.

Patient 13 was a 76-year-old woman with multiple comorbidities, including hypertension, renal failure, heart failure, and atrial fibrillation. Her treatment regimen consisted of prednisone (50 mg/day), Py (240 mg/day), and mycophenolate mofetil (1.0 g/day). Prior to EFG treatment, she experienced an MG exacerbation triggered by concurrent pneumonia and acute heart failure. After comprehensive risk-benefit assessment and thorough patient communication, EFG treatment was initiated alongside intensive anti-infective therapy. Although the first cycle of EFG did not result in MG symptom improvement, the patient demonstrated gradual recovery of pulmonary infection and cardiac function, with no infection exacerbation following EFG administration. Subsequently, the treatment regimen was transitioned to complement inhibitors. At the last visit, clinical improvement was evident, with the MG-ADL score reduced to 3 points and prednisone dosage tapered to 30 mg/day.

## Discussion

In this study, we have emphatically evaluated the efficacy of EFG in a generalized VLOMG cohort from China. We found that EFG could rapidly improve symptoms of patients with VLOMG, whether the initial status was new-diagnosed or worse. From the perspective of safety, no adverse drug reactions were observed in those patients. Furthermore, since EFG served as a fast-acting treatment for VLOMG with worsening symptoms and as an EFT therapy for new-diagnosed VLOMG, the symptoms of responded patients remained stable regardless of the maintenance treatment employed, including the use of Py alone.

Patients with MG can be categorized into three distinct subgroups based on the age of onset: early-onset MG (EOMG), LOMG, and VLOMG (25). Among the three subgroups, the proportion of VLOMG is the lowest, but this group of patients experiences a higher frequency of MC at the onset and often presents with complex complications, including hypertension, diabetes, osteoporosis, and others (9). However, the opinions on the prognosis of VLOMG were different. Cortés-Vicente and Joy Vijayan reported that VLOMG patients often had good long-term prognosis, but a cross-sectional study from China reported that elderly patients exhibited a poor prognosis and experienced a higher incidence of deaths associated with MG (3, 7, 26). Thus, personalized and fast-acting treatment at the onset of disease are very important for those patients. The usage of steroids may be limited in VLOMG patients. Among non-steroid immunosuppressants, tacrolimus was reported to be an effective and safe treatment in VLOMG, and it could be used as an initial and maintenance treatment for VLOMG (25). However, it still existed risk of hyperglycaemia, liver and renal dysfunction.

In recent years, the explosively emergence of novel biologic agents, including B-cell targeted monoclonal immunoglobulin, FcRn inhibitors, and complement inhibitors changed the treatment strategy for MG and provided more faster, more effective, and less adverse effects choices for patients with MG (20, 27, 28). EFG is an engineered human IgG1 Fc fragment designed to inhibit FcRn function, thereby decreasing the circulation of IgG and hastening its degradation (15). The phase 3 trail and its open-label extension study, as well as *post-hoc* analysis showed the fact efficacy and safety of EFG in gMG patients (15, 29–32). Thus, EFG is the first FcRn inhibitor approved for anti-AChR antibody-positive gMG in China. Recently, the first multi-center study real-world study in China was reported (24). This study enrolled more thymoma-associated patients compared with the ADAPT trial and evaluated the effectiveness of EFG in different subgroups of MG according to initial status at admission (24). However, no study focuses on the clinical application of EFG in different age onset groups so far. Thus, our study firstly evaluated the efficacy of EFG in patients with VLOMG. In comparison with the patients in the ADAPT trial, the MG-ADL score (8.2 ± 2.7) and QMG score (13.9 ± 3.6) in our cohort were slightly lower. The proportion of MG-ADL responders (defined as a reduction of MG-ADL score ≥2 points, sustained for at least 4 consecutive weeks) in AChR-gMG cohort from the ADAPT trial was 44/65 (67.7%) (15). Although our study did not use this definition due to our patients started the sequential treatment after week 5 to smoothly control symptoms, 11/15 (73.3%) of patients with VLOMG achieved CMI by week 1 and the CMI rate increased to 13/15 (83.3%) by week 5. The results suggesting a better efficacy of EFG in patients with VLOMG. The proportion of MSE in our cohort (6/15, 40.0%) was similar with those in the ADAPT trial (26/65, 40.0%) (15). Besides, the proportion of CMI and MSE were higher in new-diagnosed group, suggesting EFG to be a fast and effective therapy in those patients. In addition, consistent with the *post hoc* analyses of the phase 3 pivotal ADAPT study, our results also verified that the clinical improvements in MG-ADL total scores resulted from improvements across all subdomains (31). We found that MG-ADL scores were significantly and synchronously reduced in the ocular, limbs, and bulbar/respiratory in all patients. Although not all MG-ADL subdomains score changes were statistically significant when we analyzed different subgroups of patients, the absolute value of MG-ADL score showed a significant downward trend. It may be due to the small sample size. Thus, further studies involving larger cohorts are necessary. Additionally, two patients in this study exhibited a poor response to EFG. The reasons for this were multifaceted. Firstly, patient 6 experienced two crises, and patient 13 suffered from an infection and heart failure prior to EFG, making both patients’ disease states relatively more complex. Moreover, both patients were successfully treated with complement inhibitors in the end. This suggests that the activation of the complement system may play a more significant role in the development of disease in patients who do not respond well to EFG. However, this necessitates further in-depth research.

As we mentioned, patients with VLOMG have a high risk of myasthenic crisis at onset, so EFT was crucial for this special subgroup. The concept of EFT was initially reported in 2017. The study indicated that achieving MM or better with prednisolone ≤5 mg/day occurred more frequently and earlier in the EFT group compared to the non-EFT group (11). A more extensive study also indicated that EFT was effective for various types of MG, and the incorporation of IVMP led to earlier and more frequent attainment of MM status with prednisolone doses of ≤5 mg/day (12). However, those studies did not concentrate on the effectiveness of varying age groups at onset. Besides, the EFT strategies in these two studies were PLEX, IVIg, and IVMP, which are commonly restricted in their application to the elderly population due to the complexity of adverse drug reactions. Thus, our study tried to explore the usage of EFG as an EFT in new-diagnosed VLOMG patients. Our study included seven new-diagnosed patients with VLOMG, revealing that 7/7 (100%) attained CMI within five weeks following the initiation of EFG. Though two patients (patient 1 and patient 12) were simultaneously treated with steroids or combined with tacrolimus, but generally we think that oral low doses of steroid and tacrolimus have an effect time of longer than 2 weeks. In addition, in new-diagnosed patients with mono-Py, 1/5 (20.0%) of patients reached MSE and 5/5 (100.0%) patients reached CMI by week 5. These results highlighted that EFG could rapidly and effectively improve the symptoms of new-diagnosed VLOMG. Additionally, their condition remained stable during long-term follow-up after a single cycle of EFG, with no exacerbation noted. Therefore, the rapid reduction of disease activity in VLOMG patients with EFG at the early stage of the disease could more stably control the symptoms. This use is similar to rescue therapies, such as PLEX and IVIg. Furthermore, no patient-reported adverse events were reported in our study. However, since the adverse events were collected through interviews and self-reported by patients, there may be a possibility of underreporting of adverse events in this study. Some adverse events, such as headaches and nausea, were easily ignored according to patients’ reports. But in general, we deemed that EFG had the potential to serve as an effective and safe early fast-acting treatment option for patients with VLOMG according to our results.

This study has several limitations. Firstly, the follow-up period was short, and the number of patients who received multiple cycles of EFG was limited. Therefore, a well-designed prospective study with a long-term follow-up is needed to further demonstrate the value of EFG in patients with VLOMG. Besides, this is a single-center retrospective study with a small sample size. More multi-center studies and randomized controlled trial were needed to confirm our findings. Finally, patients were not examined for leukocyte, triglyceride or cholesterol in this study and more rigorous monitoring of adverse effects is needed.

## Conclusion

This study provided the efficacy and safety of efgartigimod in a Chinese single-center VLOMG cohort. Furthermore, this study suggests the potential of efgartigimod as a choice of early fast-acting treatment in VLOMG patients. With the continuous emergence of biological targeting agents, further prospective randomized controlled studies and longer follow-up periods are needed to examine our findings.

## Data Availability

The original contributions presented in the study are included in the article/supplementary material. Further inquiries can be directed to the corresponding authors.
